# Performance-aware programming for intraoperative intensity-based image registration on graphics processing units

**DOI:** 10.1007/s11548-020-02303-y

**Published:** 2021-01-23

**Authors:** Martin C. W. Leong, Kit-Hang Lee, Bowen P. Y. Kwan, Yui-Lun Ng, Zhiyu Liu, Nassir Navab, Wayne Luk, Ka-Wai Kwok

**Affiliations:** 1 Department of Mechanical Engineering, The University of Hong Kong, Pok Fu Lam, Hong Kong; 2 Department of Computing, Imperial College London, London, SW7 2AZ UK; 3 Computer Aided Medical Procedures and Augmented Reality, Technischen Universität München, 85748 Munich, Germany

**Keywords:** Demons algorithm, Image-guided treatment, Non-rigid registration, Parallel computing, Surgical guidance

## Abstract

**Purpose:**

Intensity-based image registration has been proven essential in many applications accredited to its unparalleled ability to resolve image misalignments. However, long registration time for image realignment prohibits its use in intra-operative navigation systems. There has been much work on accelerating the registration process by improving the algorithm’s robustness, but the innate computation required by the registration algorithm has been unresolved.

**Methods:**

Intensity-based registration methods involve operations with high arithmetic load and memory access demand, which supposes to be reduced by graphics processing units (GPUs). Although GPUs are widespread and affordable, there is a lack of open-source GPU implementations optimized for non-rigid image registration. This paper demonstrates performance-aware programming techniques, which involves systematic exploitation of GPU features, by implementing the *diffeomorphic log*-*demons* algorithm.

**Results:**

By resolving the pinpointed computation bottlenecks on GPU, our implementation of *diffeomorphic log*-*demons* on Nvidia GTX Titan X GPU has achieved ~ 95 times speed-up compared to the CPU and registered a 1.3-M voxel image in 286 ms. Even for large 37-M voxel images, our implementation is able to register in 8.56 s, which attained ~ 258 times speed-up. Our solution involves effective employment of GPU computation units, memory, and data bandwidth to resolve computation bottlenecks.

**Conclusion:**

The computation bottlenecks in *diffeomorphic log*-*demons* are pinpointed, analyzed, and resolved using various GPU performance-aware programming techniques. The proposed fast computation on basic image operations not only enhances the computation of *diffeomorphic log*-*demons*, but is also potentially extended to speed up many other intensity-based approaches. Our implementation is open-source on GitHub at https://bit.ly/2PYZxQz.

## Introduction

Image registration is a fundamental process in medical image analysis that provides accurate alignment of two image datasets. In particular, *non*-*rigid* image registration allows alignment even with the presence of uncertainties such as image distortion, physiological deformation, and different imaging modalities. Common clinical applications include anatomical atlas reconstruction [1], preoperative (pre-op) surgical planning [2], and radiotherapy dose evaluation [3]. Non-rigid registration can also facilitate image-guided interventions by realigning deformations of soft, pliable tissue that is affected by gravity, motion, and tissue–tool interaction [4, 5]. Through intraoperative (intra-op) registration, clinically valuable information from pre-op images, such as the surgical roadmap and segmented critical/target areas, can be augmented on rapidly acquired intra-op images [5]. The importance of intra-op registration can be seen in MRI-guided cardiac electrophysiology (EP) [6], where the rapid motion of the myocardium can lead to significant pre- and intra-op image misalignment. By intra-operatively registering the two image sets, the surgical roadmap can be integrated with the real-time electro-anatomical mapping to facilitate complete pulmonary vein isolation [7, 8]. However, despite the quick and high-resolution imaging of modern imaging systems, reliable intra-op registration is still computationally intensive, often requiring time in the order of minutes. To be clinically practical, the time for registration should be kept below 10 s right after each EP ablative lesion created [9]. This would allow for simple integration into the surgical workflow, and provide surgeons with up-to-date physiological information, e.g., ablation progress.

To date, many registration toolkits employ geometry-based registration, which relies on detecting, matching, and aligning image features to register the image datasets. These approaches require significant computation time and can have difficulty with feature detection due to high levels of noise and artifacts in intra-op images, leading to inaccurate registration [10]. Intensity-based registration method, such as the *Demons* algorithm which utilized the pixel values to realign a moving image on a static image, is relatively robust for non-rigid image registration [7]. Effort has been paid to further improve registration efficacy by improving the *demons* algorithm’s convergence and robustness. For example, the multi-resolution approach was used to improve the algorithm’s performance in registering highly deformed images [11]. A number of studies also focused on improving the algorithm’s efficacy by remodeling the “demon’s forces” [12] and the regularization methods [13]. Diffeomorphism was also later introduced to improve the algorithm by preserving image topology at large deformation [14].

Despite the improved robustness of intensity-based algorithms, they are generally slower than geometry-based methods due to the intensive, iterative calculations involved. In addition, the aforementioned improvements to registration efficacy often come at the cost of additional computation time. For example, the diffeomorphic *demons* algorithm [15] required > 2 min to register a brain image with 10-M voxels using two 2.8 GHz quad-core Xeon processors. The successor of diffeomorphic *demons*, the diffeomorphic log-demons, even reported that it required twice the computation although being much more robust and accurate [16].

The primary cause of these extended processing times is the computationally expensive voxel-wise operations (e.g., convolution and interpolation) requiring high-throughput, repeated memory access performed in each *demons* iteration. The resultant computation demand can even exceed the handling capability of conventional central processing units (CPUs), which are not optimized for high-throughput computation. On the other hand, graphics processing units (GPUs) excel at performing high-throughput computation due to their parallel and scalable hardware microarchitecture. Several GPU implementations of intensity-based registration are reported in [17–19], but their registration speed cannot meet the demand of time-critical surgical scenarios. For instance, the GPU implementation of 2D diffeomorphic demons reported in Huang [18] only yielded a 20 times speedup compared to an obsolete Pentium 4 CPU, and the GPU implementation of *ezys* (which belongs to a wide class of diffeomorphic demons) still required > 35 s to register a 3D image dataset at 5-megapixel resolution. To our knowledge, there are no reported implementations of intensity-based algorithms that can fulfill the strict time constraints for intra-op registration.

To this end, we propose a GPU-based optimization framework to minimize the runtime for intensity-based non-rigid registration. We apply *performance*-*aware programming* [20], which is a key method to achieving high-throughput, low-latency computation, through systematic exploitation of the device and the algorithm. It involves the effective employment of different GPU resources, such as computation units, memory, and data bandwidth to resolve and avoid computation bottlenecks. These optimization techniques are demonstrated on the well-known *diffeomorphic log*-*demons* [16] algorithm. This algorithm is chosen as a benchmark not only because of its reliability and accuracy, but also because it comprises basic image operations, namely interpolation and convolution, that are ubiquitous in many other registration algorithms such that the performance-aware optimizations employed can be translatable. The main contributions of this work are:

- Pinpointing, analyzing, and resolving the computation bottlenecks of intensity-based non-rigid registration using various GPU performance-aware programming techniques;
- Quantifying the computation enhancement by comparing the optimal and sub-optimal GPU implementations of the bottlenecking operations; and
- Providing an *open*-*sourced* library with optimal GPU implementation of diffeomorphic log-*demons*.

## Materials and methods

### GPU-based performance-aware programming

*Performance*-*aware programming* is an iterative process alternating between profiling and optimization on the bottlenecking operations. These computation-intensive operations could impose a significant challenge in time-critical applications. This process requires understanding on the bottleneck operation of an algorithm, the existence of such bottlenecks, and the corresponding techniques to overcome this barrier. Therefore, performance-aware programming optimizes thread/warp utilization, resource/register allocation and memory access patterns for the best possible performance enhancement.

**Fig. 1:**
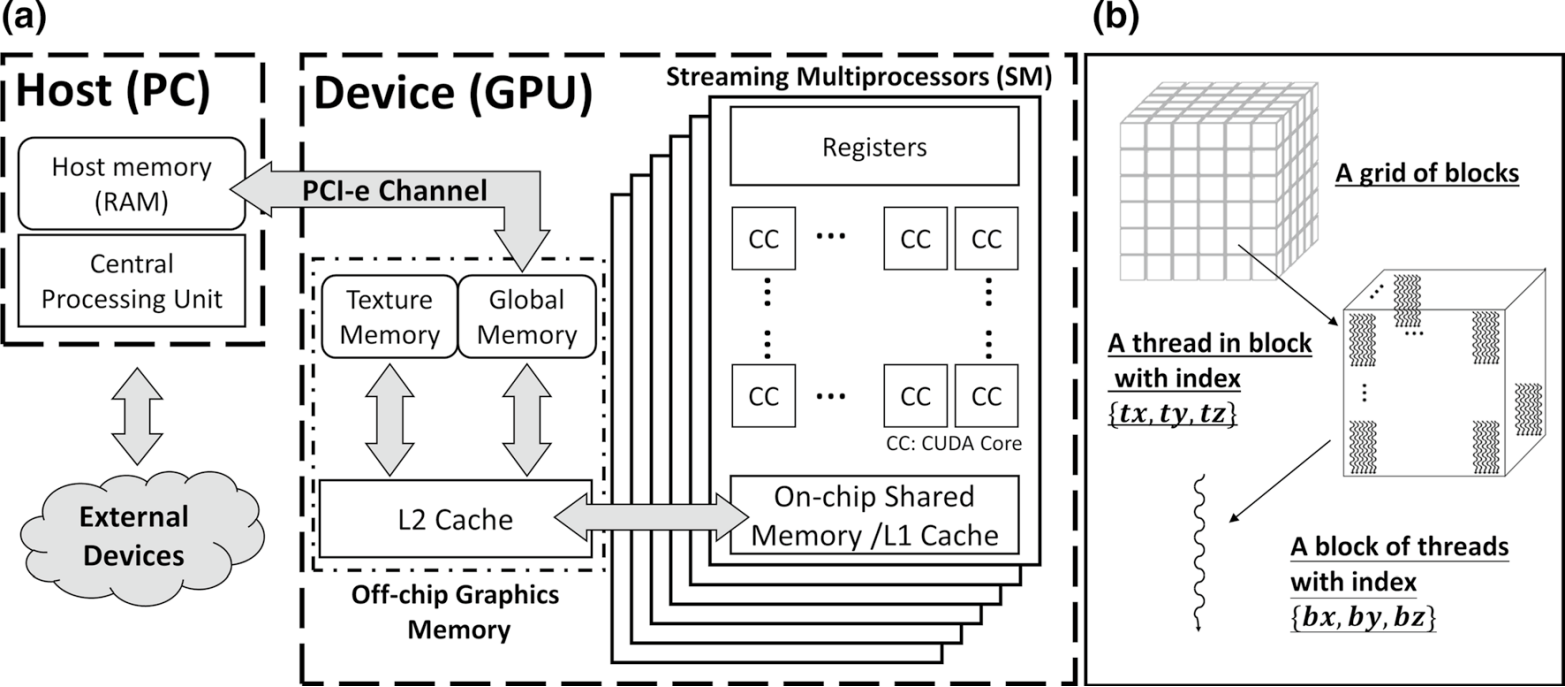
Simplified schematic diagram illustrating CUDA hardware and software architecture. **a** CUDA GPU possesses numerous streaming multiprocessors as its basic computation units. The streaming multiprocessors can access the off-chip graphics memory via a heavily cached data bus. **b** Upon kernel execution, a grid of thread blocks which consist of numerous threads are instantiated

GPUs are specialized hardware originally developed for rendering images in a highly parallelized manner. A general hardware microarchitecture of a CUDA-compatible devices (CUDA device) is presented in Fig. 1a. The basic computation unit of a CUDA device is the streaming multiprocessor (SM), which possesses many *CUDA cores*. SMs are capable of processing concurrent threads efficiently under the *Single Instruction, Multiple Threads (SIMT)* architecture (Fig. 1b). In view of achieving high-performance, the 3 essential features of CUDA GPUs are: (**Feature 1**) support of highly parallel computation; (**Feature 2**) efficient memory caching through the user-managed cache; and (**Feature 3**) dedicated texture hardware.

**Fig. 2:**
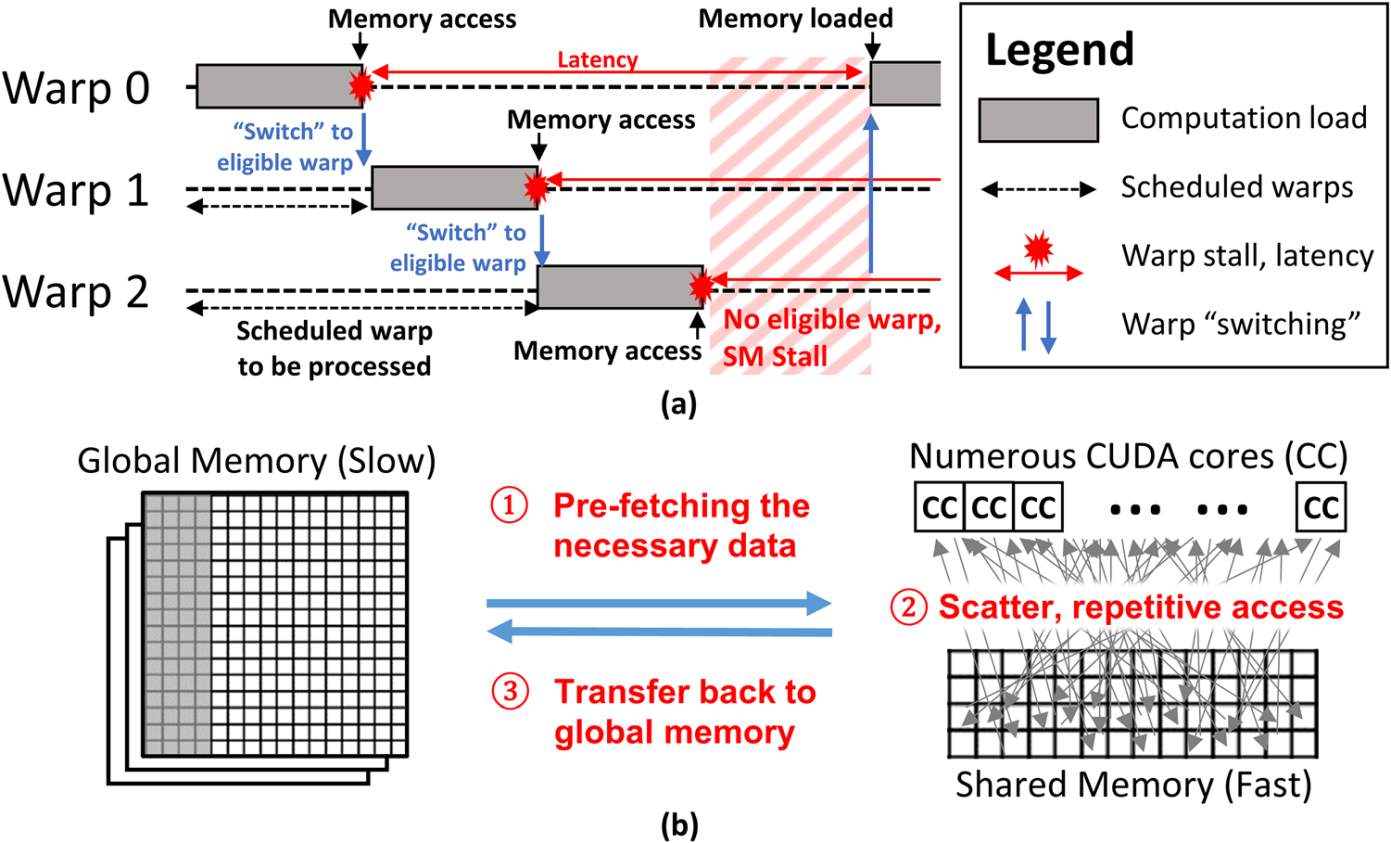
Essential features of the GPU to achieve high-performance computing. **a** The SM can execute warps of 32 threads in parallel under SIMT; latency can be mitigated by the SM’s ability to “switch” between eligible warps for concurrent execution. **b** Memory access bottlenecks to the global memory can be avoided by ① pre-fetching the necessary data onto the faster shared memory for ② scatter or repetitive access. The computation results can be ③ efficiently transferred back to the global memory once completed

**Feature 1** is achieved by the dedicated microarchitecture of SM which executes the threads in a highly parallel manner. Under the SIMT architecture, the CUDA cores execute *warps* of 32 threads simultaneously under a single fetch-decode instruction cycle (Fig. 2a). The maximum number of warps executable on an SM, known as occupancy, is determined by the kernel’s demand of computation resources such as registers and shared memory. As such, optimizing the SM’s occupancy by managing the resources is often important.

**Feature 2** can be achieved by the efficient usage of the on-chip shared memory on each SM. As accessing the shared memory is much (> 80×) faster than the global memory, the shared memory can act as an efficient, user-managed cache to temporary store any data for computation (Fig. 2b). Effective use of shared memory can reduce both global memory bandwidth and register pressure [21].

Finally, CUDA GPU can benefit from **Feature 3** which is dedicated to optimizing the texture filtering (interpolation) process. As normal 3D interpolation is both arithmetic and memory intensive, the GPU’s texture hardware is designed to efficiently complete the computation through its optimized hardwired data channels. Thus, the 3D interpolation calculation can be accelerated by the texture hardware with the trade-off of reduced interpolant precision.

### Performance-aware optimizations

This section describes applying performance-aware optimizations to intensity-based registration, systematically exploiting the three GPU features identified in Section II. Most *demons*-based registration algorithms contain complex and iterative operations, such as deformation based on concepts of optical flow with small steps. A substantial (typ. > 50) amount of *demons* iteration is therefore required to complete the registration, especially when there is large misalignment between the images.

**Fig. 3:**
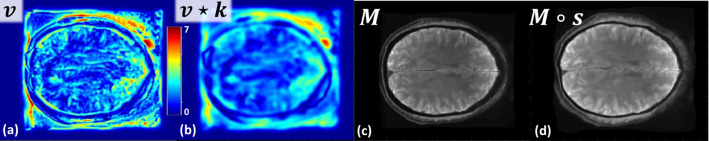
Two bottleneck operations in the *diffeomorphic log*-*demons* algorithm. **a**, **b** Convolution (⋆) of a velocity vector field (*v*) by a Gaussian kernel (*k*); **c**, **d** Warping (∘) of a is moving image (*M*) by a deformation field (*s*) Source of sample image data: The Cancer Imaging Archive (TCIA)

We have identified the key computation bottlenecks of *diffeomorphic log*-*demons* (Algorithm 1), such as vector field regularization and diffeomorphic field mapping, which are also prevalent in many other intensity-based registration schemes. Each iteration of *diffeomorphic log*-*demons* consists of multiple convolution (⋆) and composition/warping (∘) operations (Fig. 3). Particularly, computation of the deformation field using the *scaling and squaring method* (*line* *7a*-*d* in Algorithm 1) consists of repeated vector field self-composition. Regularization of vector field via convolution can also be costly. Profiling on the open-sourced MATLAB implementation^1^ showed these two operations take up ~ 85% of CPU computation time. Therefore, the computation bottlenecks in (1) *vector field regularization* (*line* *4 & 6* in Algorithm 1) and (2) *image warping & field composition* (*line 5, 7d & 8 in* Algorithm 1) will have to be resolved.
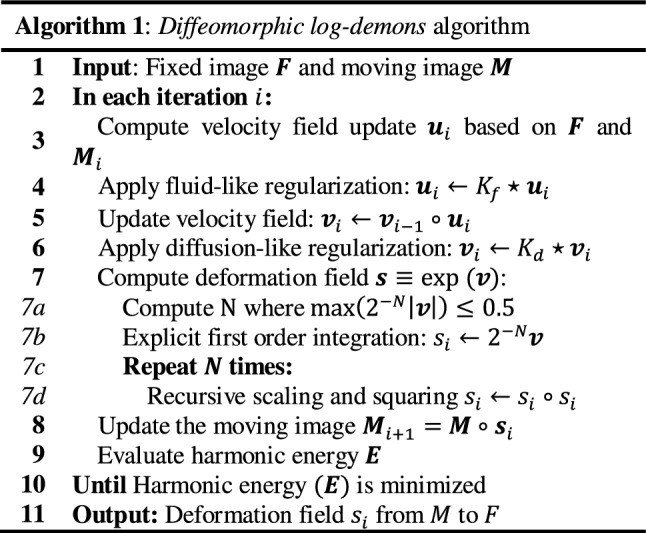

### Optimizing vector field regularization

Gaussian filtering (“Gaussian kernel”) is extensively used on vector fields as a simplified model of deformation propagation [22] and regularization [13]. However, a naïve implementation of 3D Gaussian filtering requires access to nearby $\left( {6\sigma } \right)^{3}$ elements for each voxel, involving numerous data transactions, especially when $\sigma$ is large. To this end, it is common to decompose the demanding 3D convolution into three 1D convolutions. The formulation of 1D Gaussian kernels ($k\left[ d \right]$) is shown in (1). The workflow of performing multi-pass 3D filtering by a symmetrical kernel on a vector field is presented in Algorithm 2.
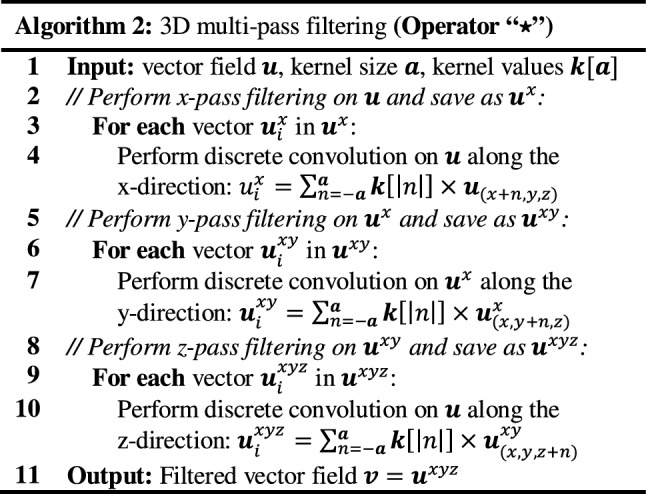

1 $$ k\left[ d \right] = \frac{1}{{\sigma \sqrt {2\pi } }}\exp \left( { - \frac{{d^{2} }}{{\sigma^{2} }}} \right), d = \left\{ {0,1, \ldots ,{\text{nint}}\left( {3\sigma } \right)} \right\} $$

**Fig. 4:**
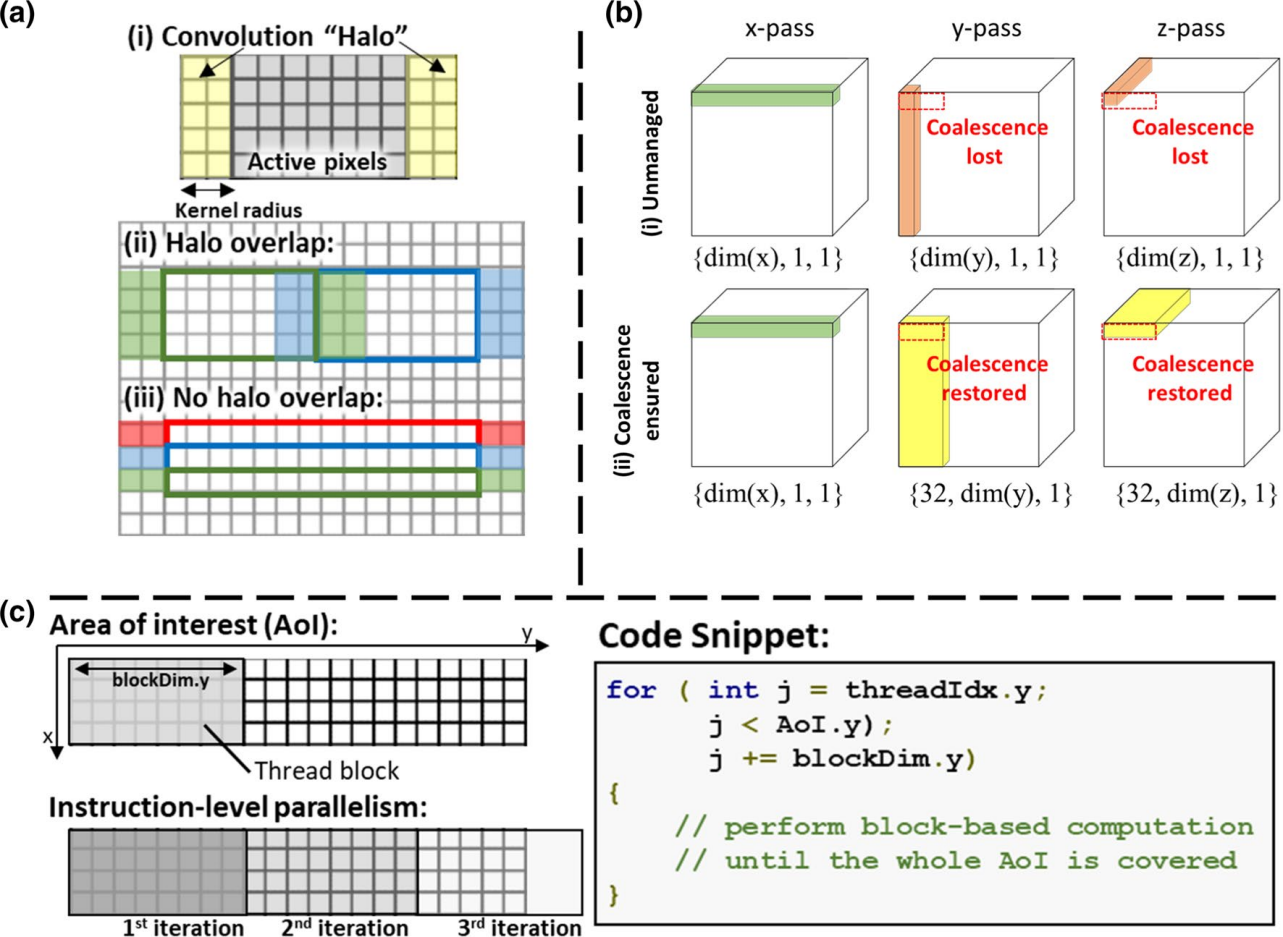
Essential performance-aware programming techniques for optimizing the convolution operation on a vector field. **a** The convolution operation requires loading additional memory items in “halo” regions around the active pixels for computation for each thread block. Redundant memory access due to overlapping of the halo regions by the blue and green thread blocks can be mitigated by instantiating suitable block dimensions. **b** Slander blocks along the *y*- and *z*-direction can cause the loss of memory coalescence (thread blocks in red), which can be rectified by instantiating appropriate *x*-dimension (blocks in yellow). **(c)** Instruction-level parallelism can be used to allow the treads to be re-used to cover the whole area-of-interest in an iterative manner. Such technique is useful in reducing the overall number of threads in compliance to the GPU hardware limitation

*x*-*pass filtering* The voxel-independency of multi-pass Gaussian filtering facilitates the use of **Feature 1** for parallel computation. To alleviate memory contention, the shared memory on the SMs (**Feature 2**) can be used to actively pre-fetch and reuse the data that are nearby spatially localized. However, as illustrated in Fig. 4a(i), the convolution operation requires access to the active pixels in addition to its local neighbor memory elements (the *“halo”*) for computation. Overlapping of such “halo” results in redundant global memory access which undermines efficiency (Fig. 4a(ii)). This overlapping can be eliminated by instantiating thread blocks and shared memory with size equal to *x*-dimensions of the image (Fig. 4a(iii)).

*y/z*-*pass filtering* Pre-fetching data requires striding the memory which the L2 cache will often struggle to handle. Furthermore, instantiating the thread blocks and shared memory with size equal to the *y*-*/z*-dimensions will result in bandwidth under-utilization due to the lost of 128-byte memory coalescence (Fig. 4b(i)). To assign appropriate *x*-dimensions to the thread blocks is important to restore the 128-byte coalescence (Fig. 4b(ii)). However, such strategy increases the required number of threads per block by 32-fold which can exceed the hardware limit imposed by the SM. To this end, instruction-level parallelism can be utilized, which allows a single thread block to cover the whole area-of-interest in an iterative manner (Fig. 4c).

Finally, the memory latency associated with pre-fetching necessary data onto the shared memory can be further reduced by micro-optimizations such as temporarily casting the variables into vectorized format (e.g., *float4*). Fetching vectorized data facilitates the generation of more efficient PTX instructions. For instance, loading a *float4* vector from the global memory can be achieved by a single *ld.global.v4.f32* instruction, which is faster than executing multiple *ld.global.f32* instructions.

### Optimizing image warping and vector field composition

Warping/composition operations (Algorithm 3) are mandatory in the *diffeomorphic log*-*demons* algorithm for obtaining the intermediate moving image in each iteration. Vector field composition (Algorithm 4) is essential for updating the velocity field and deformation field. Both operations share the same operator (∘) which applies a deformation field through trilinear interpolation.
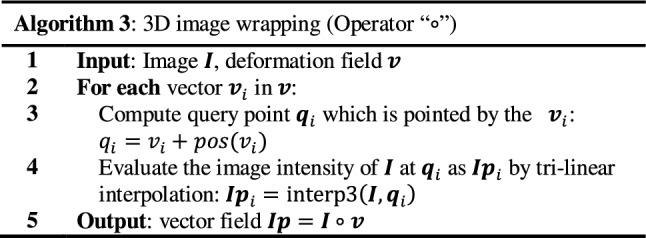

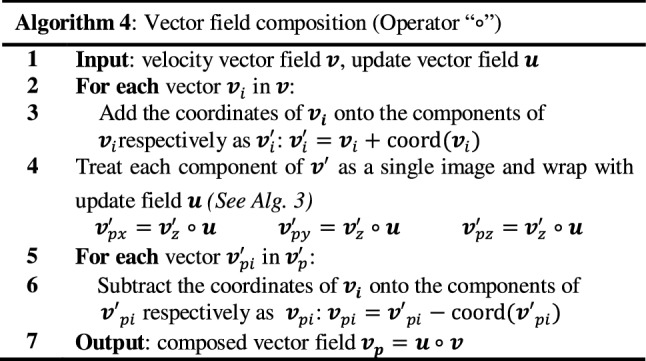


The inherent voxel independency of the image warping and vector field composition makes the algorithm highly parallelizable. However, warping an image by a nonparametric vector field implies inevitable scatter, cache-unfriendly memory access. As such, only a fraction of the 128-byte coalesced data batch may be useful to the SM, which can induce tremendous latency. Worse still, trilinear interpolation requires data from the 8 nearby elements around the query point. These elements, despite located in 3D proximity, are separated by large strides on the 1D memory array. As a result, attempts to fetch nearby data along the *y*-*/z*-directions will incur extra latency due to cache misses.

The GPU can also utilize its underlying texture hardware (**Feature 3**) to improve memory access performance. Above all, the GPU’s texture cache excels in fetching 3D spatially localized elements. The hardware managed “pitched” pointers are also capable of resolving any potential memory misalignments. This feature is particularly useful because the texture references can be updated to allow fast read-only access to the 3D spatially localized elements.

### Optimization to other parts of computation

Albeit not being the major computation bottleneck, many other operations in *diffeomorphic log*-*demons* are also eligible for GPU acceleration. For example, optimizing SM occupancy ensures the full use of the GPU. Enforcing memory alignment with respect to the 128-Byte L1 cache can be beneficial to all Global-L1 memory transection, which can be further optimized by temporarily type-casting an array into vectorized variables [23]. The use of the keyword, “*__restrict__*”, may allow the compiler to generate more efficient codes that disregard any provision to the *pointer aliasing* [24] issue, as the keyword guarantees no overlap between separate memory segments. Other optimization such as resolving thread divergence and utilizing the GPU’s hardwired functions by enabling the –*use_fast_math* compiler flag can also yield additional improvement.

## Results

We quantify the performance enhancement brought by *performance*-*aware programming* techniques by comparing different implementations of the bottleneck operations in *diffeomorphic log*-*demons*. All experiments are performed using a PC equipped with Intel i7-4790 CPU (3.6 GHz) and a Nvidia GTX Titan X GPU. The code was compiled in the -O3 optimization level for higher performance [25]. A collection of sample brain MRI image is acquired from The Cancer Imaging Archive (TCIA) [26] for the benchmarking purpose. The velocity vector fields used for the experiment is obtained by registering a pair of pre-/post-deformed brain MRI image using *diffeomorphic log*-*demons*. The effect of varying input dimension is investigated by up/down-sampling the vector fields into 7 levels of resolutions, ranging from 1.3 to 37-M voxels. To avoid host-device memory transaction interfering with the results, all necessary data are transferred to the GPU graphics memory prior to the experiments.

### Optimizing vector field regularization

**Feature 1** and **Feature 2** are used to optimize the vector field regularization. To systemically evaluate the effectiveness of these optimizations, three implementations of Gaussian smoothing on a vector field are developed, namely:

1. Naïve implementation without any data reuse;
2. Employing data reuse by the shared memory; and
3. Initializing the shared memory with coalesced global-shared memory transactions.

The variance of the Gaussian kernel is set to 3, which is a typical value for regularization. Among the 3 implementations, A1 is the naïve implementation that solely utilizes the GPU’s ability of parallel processing (**Feature 1**). A2 and A3 attempted to make use of the shared memory (**Feature 2**) for effective caching; however, A2 does not consider coalesced memory transaction during the initialization (Fig. 4b(i)). A3 ensures memory coalescence during the initialization by assigning appropriate block dimensions (Fig. 4b(ii)).

**Fig. 5:**
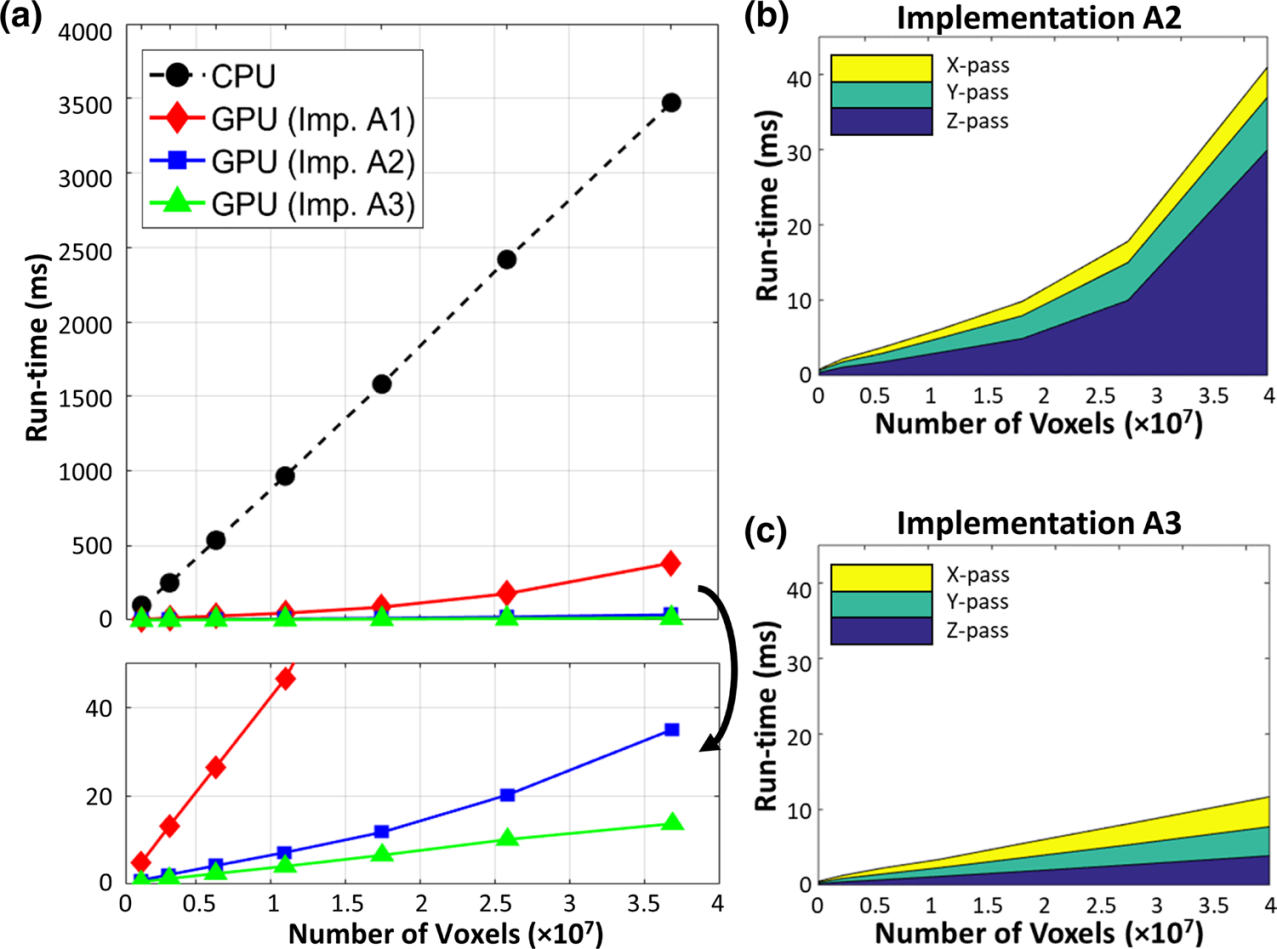
**a** Run-time required for CPU and the three different GPU implementations in order to compute the convolution on a vector field at 7 levels of resolution. **b**, **c** Breakdown of run-time of implementations, Imp. **A2** and **A3**, required to complete the *x*-/*y*-/*z*-passes of the convolution. Convolution at the z-direction takes up most runtime especially at high resolution

Figure 5a presents the time required for the single-threaded CPU and the 3 GPU implementations to complete the regularization process. The CPU requires 100 ms to complete the smoothing computation for the smallest 1.3-M voxels vector field, and 3500 ms to complete a large vector field (37-M voxel). In contrast, the GPU can perform the computation significantly faster than the CPU. Implementation A1 achieves the same computation within 5 to 500 ms. The performance further increases significantly when data reuse techniques are employed. The optimal implementation, A3, can complete the computation within 0.9–13.5 ms, which is 170–250 × faster than the CPU.

**Fig. 6:**
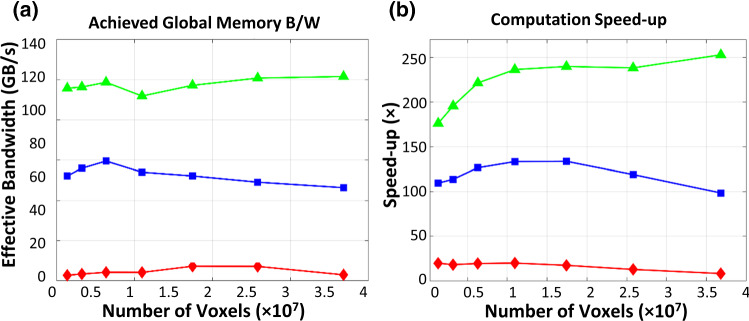
**a** Achieved global memory bandwidth, and **b** resulting speed-up achieved by the three different GPU implementations

Despite both A2 and A3 make uses of the shared memory as a user-managed cache for fast Gaussian convolution, the two implementations show a large disparity in terms of performance. Implementation A2 struggles in performing the *z*-pass filtering (Fig. 5b, c), particularly at high vector field dimension. As the vector field dimensions increases, a larger memory stride will be required fetch data across the *z*-direction. For example, at 26-M (278 × 334 × 278) voxels, memory strides of 278 × 334 × size of (float) = 363 KB is required. As the L2 size on each memory controller of the Titan X GPU is only 512 KB, the L2 hit rate decreases drastically at high dimensions. The caching efficiency can be improved by ensuring coalesced global memory transaction as in implementation A3. Despite striding is still required, coalesced data can be fetched simultaneously without the need of issuing additional read instruction. This can improve the bandwidth utilization, which can be reflected by the achieved global memory bandwidth and speed-up (Fig. 6a, b).

### Optimizing image warping/vector field composition

The GPU can utilize **Feature 1** and **Feature 3** to accelerate image warping and vector field composition. Performance enhancement of vector field composition will be studied, as it is an extension of warping. Similar workflow will be adopted in the evaluation of the four implementations to perform the composition, namely:

1. Kernel interpolation on the global memory;
2. Kernel interpolation on global memory via the texture data paths;
3. Hardwired interpolation using the texture hardware; and
4. Hardwired interpolation on vectorized elements.

Implementation B1 is the naïve implementation of the vector field composition and only utilizes the parallel computation power GPU (**Feature 1**) to perform the interpolation. Implementations B2–B4 utilize the GPU’s texture hardware (**Feature 3**) to accelerate the computation. First, implementation B2 makes uses of the texture data path to fetch the 3D spatially localized elements for interpolation. Implementation B3 further utilizes the texture filtering hardware by accessing the *cudaTextureFilterMode* option in the texture descriptor for fast interpolation. Finally, implementation B4 extends B3 by fetching and filtering the elements in a coalesced manner u*s*ing vectorized data types (i.e., *float4*).

**Fig. 7:**
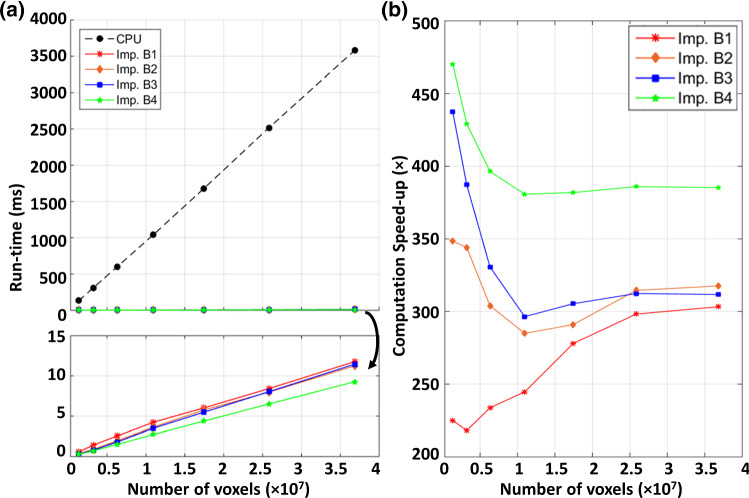
**a** Run-time required for CPU and the four different GPU implementations to compute the composition on a vector field at 7 levels of resolution. **b** Achieved computation speed-up with those fours

**Fig. 8:**
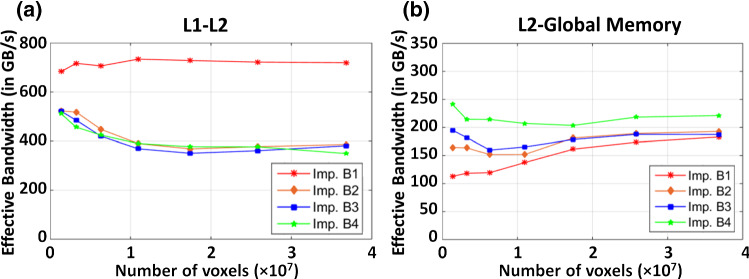
The achieved **a** L1–L2 bandwidth, and **b** L2-Global memory bandwidth by the four different GPU implementations

Figure 7a presents the comparison of computation time required to perform a vector field composition. Figure 7b presents the achieved computation enhancement by different GPU implementations. All GPU implementations achieve significant computation speed-up compared to the CPU. Implementation B1 achieves the worst performance enhancement due to the lack of memory optimization. Strided memory access required to perform voxel-wise interpolation occupy substantial memory bandwidth. Despite L2 cache can relieve the global memory bandwidth stress, numerous fetch requests from the SM can saturate the memory bandwidth between L1 and L2 caches. This can be reflected by the fact that the achieved L1–L2 bandwidth for implementation B1 remained all-time high (Fig. 8a). Thus, this high latency induced by memory contention on the L1–L2 data channel undermines the efficiency and utilization of the global memory (Fig. 8b). It is also noteworthy that the achieved global memory bandwidth increases at higher vector field dimension, which can be accounted for the latency-mitigating ability of the extra warps launched for voxel-wise interpolation.

Implementation B2–B3 utilize the GPU’s texture caches to resolve the L1–L2 memory contention and improve the L2-global memory bandwidth utilization. B3 further mitigates the arithmetic load requirement of interpolation by offloading to the dedicated texture mapping unit. However, the computation speed-up shows an interesting phenomenon: both implementations achieve better performance at lower vector field dimensions, but the performance drops and levels off at the high dimension. B2 achieves ~ 350 times speed-up; B3 achieves ~ 440 times speed-up at low resolution. However, the performance speed-up achieved by both implementations falls to ~ 320 × at high vector field dimension.

**Fig. 9:**
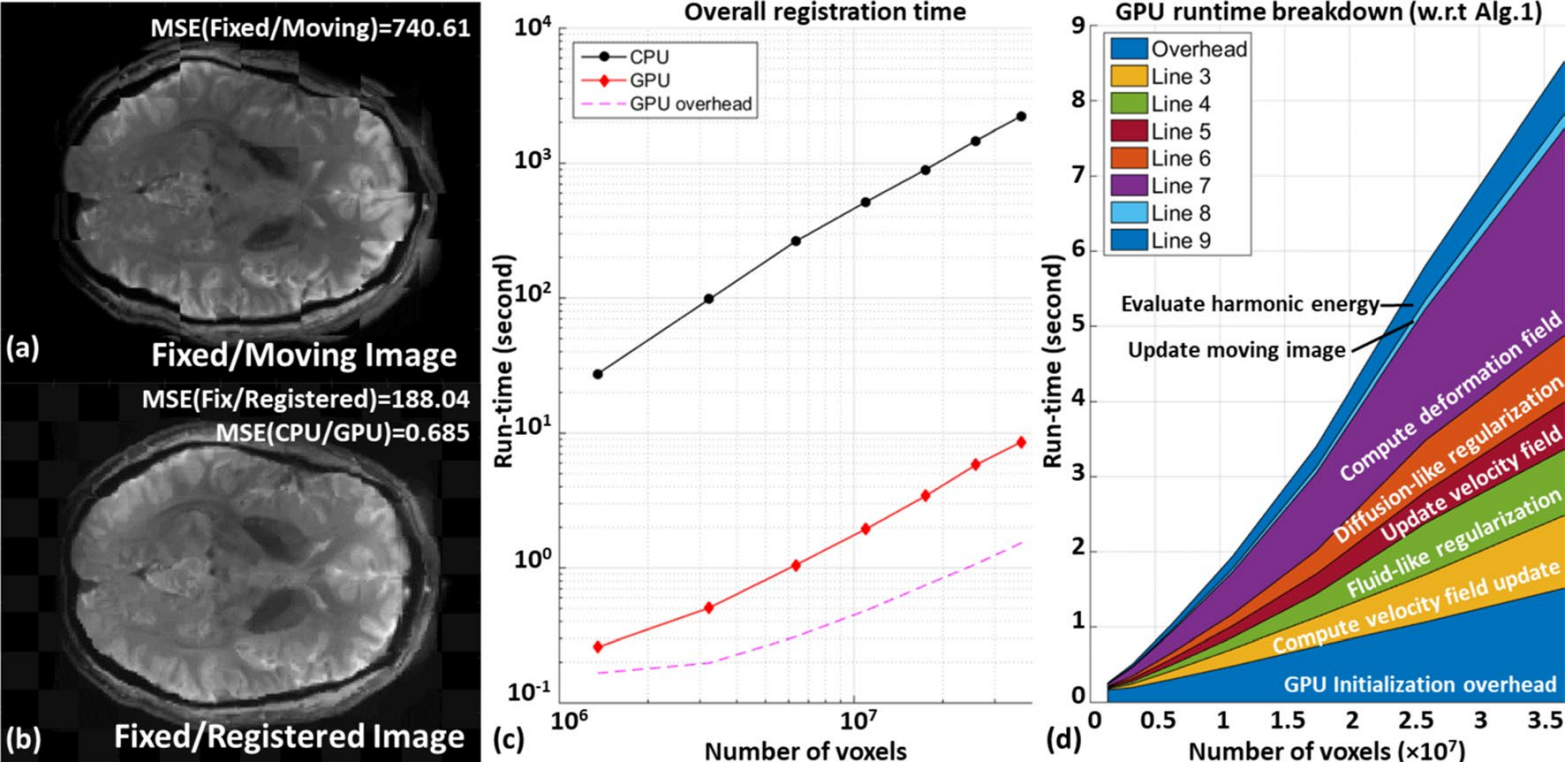
**a**, **b** Checkerboard images showing the large misalignment between the fixed and moving image pair has been resolved in the registered image. Negligible MSE between the CPU and GPU results suggests the two implementations are consistent. **c** Run-time required for CPU and our optimized GPU implementation of *diffeomorphic log*-*demons* to complete the registration. A promising acceleration with two orders of magnitude over a CPU is achieved. The GPU initialization overhead is illustrated by the dotted line in purple. **d** Breakdown of the runtime of the key computation steps as presented in Algorithm 1. Source of image data: TCIA

We hypothesize that the global memory may be limiting the computation at high dimension. It is noted that both implementations achieved higher L1–L2 bandwidth at low vector field dimension (Fig. 9b). The increased L1–L2 activity signifies high L2 hit rate. At low vector field resolution, the L2 cache is able to hold a larger portion of the vector field which mitigates global memory latencies. As the vector field dimension increases, subsequent decreases in L2 hit rate incur much more global memory transactions that cause contention on the memory controller. Such contention on the memory controller is resolved by implementation B4, which restructures the elements into an array of vectorized variable during data transections. Fetching vectorized elements reduces the number of transaction requests to the memory controller, thus alleviating the memory contention. As a result, overall higher global memory bandwidth utilization is observed, with ~ 470 times speed-up at low resolution and ~ 380 times speed-up at a higher resolution in implementation B4.

### Overall optimization results

We have implemented the entire pipeline of the algorithm with different performance-aware programming strategies, as stipulated in Table 1. As a result, our optimized GPU implementation of *diffeomorphic log*-*demons* achieved an impressive computation enhancement compared to the CPU. The GPU implementation is also validated to ensure it behaves consistently along with the CPU implementation (Fig. 9a-b). Figure 9c presents the run-time to register the MRI brain images in 7 levels of resolutions with the same parameters by CPU and GPU. The CPU requires prolonged computation to register the images. Even with the smallest dataset, namely a pair of 1.3-M voxel images, the CPU requires ~ 27 s to complete the registration. Efficient utilization of GPU reduces the registration time to 286 ms. As the dataset resolution increases, the CPU run-time gradually increases. Registering the highest resolution (37-M voxels) requires > 2200 s (36 min) which is prohibitively long for many applications. The GPU implementation is able to complete the registration of highest resolution in 8,561 ms, suggesting the computation time can be diminished with our optimized GPU implementation.

**Table 1:** Optimization strategies used in the performance-aware implementation

Computation	Bottleneck	Optimization strategies
Finite image difference	Memory	Optimized parallelization **(Feature 1)**
		Code micro-optimizations
Gradient decomposition	Memory	Optimized parallelization **(Feature 1)**
		Efficient user-managed cache **(Feature 2)**
Gaussian convolution	Memory	Optimized parallelization **(Feature 1)**
		Efficient user-managed cache **(Feature 2)**
		Code micro-optimizations
Image warping/field composition	Memory/computation	Optimized parallelization **(Feature 1)**
		Use texture hardware **(Feature 3)**
Update field computation	Computation/control flow	Optimized parallelization **(Feature 1)**
		Code micro-optimizations
Maximum/sum reduction	Algorithm	Kernel decomposition
		Code micro-optimizations

The parallel processing ability of the GPU consistently provides high computation throughput for *diffeomorphic log*-*demons*. However, host-device overhead in GPU initialization takes up substantial computation time. At low resolution, more than half of the runtime (166 ms/286 ms) is spent on allocating memory onto the GPU. At high resolution, this overhead diminishes as the computation time increases (1500 ms/8561 ms). The computation speed-up increases from ~ 95 times at low resolution to ~ 258 times speed-up at high resolution.

Figure 9d presents the time consumption breakdown by the key computation steps in the optimized GPU implementation. The time consumed by the previously identified computation bottleneck, namely vector field regularization and deformation field computation, is now comparable to other operations. The reduced computation time occupied by the two computation steps suggests the bottlenecks have been resolved.

## Discussion and conclusion

We proposed several optimized computation enhancements on intensity-based registration algorithms using various GPU performance-aware programming techniques, and quantified the enhancements by comparing the optimal and sub-optimal GPU implementations. The optimization techniques are demonstrated on a benchmark intensity-based registration algorithm, the well-known *diffeomorphic log*-*demons* algorithm, to pinpoint, analyze, and resolve the computation bottleneck in each computation step. Convolution and composition/warping are common yet slow voxel operations, which are ubiquitous in many other intensity-based registration algorithms. In this regard, we conducted comprehensive testing to optimize thread/warp utilization and memory access patterns such that the bottlenecking operations can be resolved. By utilizing appropriate GPU features, we have achieved significant acceleration: ~ 250 times for convolution and ~ 380 times for composition/warping. Overall, our GPU implementation of *diffeomorphic log*-*demons* has achieved ~ 200 times speed-up. This outstanding performance enhancement also enables the algorithm to have similar, if not shorter, registration time with many popular registration packages such as SPM [27] and elastix [28].

Having accelerated basic image operations does not only enhance the *diffeomorphic log*-*demons*, but also can potentially speed up many other demons-based approaches, such as *spherical demons* [29], *adaptive demons* [30]. However, implementing these fully optimized algorithms can involve tremendous efforts. In this regard, we have developed an open-source version of the optimized *diffeomorphic log*-*demons* which is available at GitHub. This facilitate the translation to other intensity-based registration algorithms using the similar voxel-based operations and serve as a baseline for future comparison. Our implementation is cross-platform compatible. To incorporate our work for open-source library such as ITK [31], a wrapper is also provided for DICOM images loaded in ITK to call our implementation and perform the registration. This library will also be incorporated as a module to other open-source medical imaging processing platforms, e.g., ITK [31] and 3D Slicer [32] and in the future. As many advanced intensity-based image registration algorithms share similar workflow involving voxel-wise interpolation and convolution operations, our established acceleration schemes can be translated effortlessly to boost the performance. This optimized GPU implementation also enables the use of the *diffeomorphic log*-*demons* algorithm in many time-critical applications.

## Footnotes

1 Available on: https://www.mathworks.com/matlabcentral/fileexchange/39194-diffeomorphic-log-demons-image-registration.
